# Structural basis for a novel type of cytokinin-activating protein

**DOI:** 10.1038/srep45985

**Published:** 2017-04-04

**Authors:** Hogyun Seo, Kyung-Jin Kim

**Affiliations:** 1 School of Life Sciences, KNU Creative BioResearch Group, Kyungpook National University, Daegu 702-701, Republic of Korea

## Abstract

The Lonely Guy (LOG) protein has been identified as a crucial enzyme involved in the production of cytokinins, which are important phytohormones, in plants and plant-interacting organisms. However, *C. glutamicum* has an isoform (*Cg*1261) of LOG that contains an extended N-terminal region compared to those of known LOGs, and this type of isoforms are also found in a variety of organisms. Nevertheless, these proteins are considered as lysine decarboxylases, without their functional characterization. To investigate the function of *Cg*1261, we determined its crystal structure at a resolution of 1.95 Å. Unlike known dimeric LOGs, *Cg*1261 was found to form a hexamer. The overall shape of the hexamer resembles a trillium flower, in which a twisted dimer constitutes each petal. The dimeric petal is well superposed with known LOG dimers, and its active site conformation is similar to those of LOG dimers, suggesting that the hexameric LOG-like protein also acts as a LOG. Biochemical and *in vivo* cytokinin production studies on *Cg*1261 confirms that *Cg*1261 functions as a cytokinin-activating protein. Phylogenetic tree analysis using 123 LOG-like proteins suggest that the LOG-like proteins can be categorized to the dimeric type-I LOG and the hexameric type-II LOG.

Cytokinins are plant hormones crucial for promoting cell division and differentiation in plants^1^. These molecules are chemically known as purine bases, such as isopentenyladenine (iP), *trans*-zeatin, and 6-benzylaminopurine, in which the N^6^ atom is modified with isoprenoids or aromatic rings^1^. Cytokinins often appear as conjugated forms with sugar moieties such as nucleotides, nucleosides, and glucosides, which are biologically less active or inactive for plant receptors^2^. In cytokinin biosynthesis, the Lonely Guy (LOG) protein has been recently identified as a phosphoribohydrolase that finally releases cytokinins^3^. LOG proteins produce active cytokinins via dephosphoribosylation, directly hydrolyzing the bond between N^6^-substituted bases and ribose 5′-monophosphates in conjugated forms. Importantly, the LOG domain is conserved in a wide range of organisms^3^,^4^,^5^, and the majority of LOG proteins are from prokaryotic organisms. However, bacterial LOGs have been especially poorly understood because a LOG protein was originally characterized as a phytohormone-activating plant enzyme, while LOG-like proteins have been considered possible lysine decarboxylases (LDCs) so far.

*Corynebacterium glutamicum* is widely known for its advantage in production of amino acids, nucleotides, and vitamins^6^. Although the relationships of *C. glutamicum* with plant species have not yet been reported, we have previously shown a phosphoribohydrolase activity of misannotated *Cg*2612 and proposed it as *Cg*LOG based on the structural and biochemical characteristics, which may indicate as yet undiscovered interactions of this microorganism with plants^7^. Interestingly, we have found that *C. glutamicum* has an isoform (*Cg*1261) of *Cg*LOG. *Cg*1261 contains an extended N-terminal region compared to those of known LOGs, including *Cg*LOG, and this type of isoforms have also been found in a variety of organisms. However, a protein from *Thermus thermophilus* HB8 (*Tt*1465), which is homologous to *Cg*1261, was once assigned as a possible LDC before the discovery of its LOG identity^8^, and these proteins have also been considered as LDCs, without their functional characterization^9^,^10^,^11^. Thus, the function of this new LOG-like protein from *C. glutamicum* remains totally unknown. Therefore, despite the high similarity of *Cg*1261 to other known LOGs, it is not clear whether *Cg*1261 is actually a LOG protein.

To investigate the function of *Cg*1261, we determined its crystal structure at a resolution of 1.95 Å and revealed its hexameric oligomerization involving the extended N-terminal region. Based on the biochemical and *in vivo* cytokinin production experiments, we propose that *Cg*1261 is a novel type of LOG and belongs to type II LOGs (*Cg*LOGII). Comparative analysis of 123 LOG-like proteins also suggested that LOG proteins could be divided into two different types, dimeric type I LOGs and hexameric type II LOGs.

## Results

### Monomeric and dimeric structure of *Cg*1261

**Figure 1:**
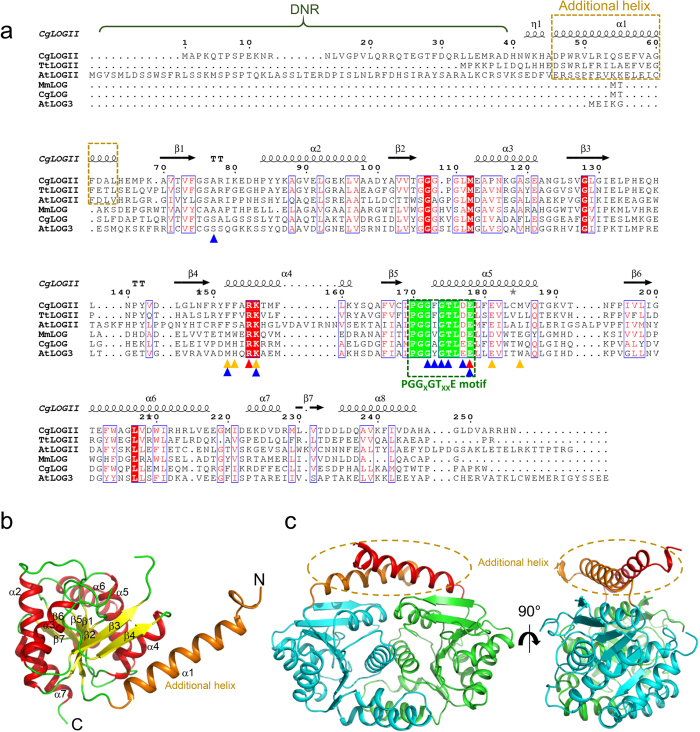
Overall structure of *Cg*LOGII. (**a**) Amino acid sequence alignment of LOGs. The figure of aligned sequences was generated by *ESPript* 3.0 software. The secondary structure elements are drawn based on the structure of *Cg*LOGII. Residues involved in enzyme catalysis, AMP binding, and prenyl-group binding are indicated by red, blue, and orange-colored triangles, respectively. The PGGxGTxxE motif and the additional α-helix from type-II LOGs are indicated with green- and orange-colored dotted rectangle, respectively. The disordered N-terminal region (DNR) is labeled. *Cg*LOGII, *Tt*LOGII and *At*LOGII are abbreviations of the type-II LOGs from *C. glutamicum* and *T. thermophiles* and *A. thaliana*, respectively. *Mm*LOG, *Cg*LOG and *At*LOG3 are abbreviations of the type-I LOGs from *M. marinum, C. glutamicum* and *A. thaliana*, respectively. (**b**) Monomeric structure of *Cg*LOGII. The monomeric structure of *Cg*LOGII is presented as a cartoon diagram. Secondary structure elements are labeled. The additional α-helix located at the N-terminal region is distinguished with an orange color and labeled. (**c**) Dimeric structure of *Cg*LOGII. The dimeric structure of *Cg*LOGII is presented as a cartoon diagram. Two additional α-helices from two monomers are indicated with an orange-colored circle and labeled. The right figure is the left figure rotated horizontally by 90°.

To elucidate the function and molecular mechanism of *Cg*1261, we determined its crystal structure at a resolution of 1.95 Å (Table 1). The asymmetric unit contained three molecules (Molecules I, II, and III), and the crystal volume per unit of protein mass was approximately 1.91 Å^3^·Da^−1^, which corresponds to a solvent content of approximately 35.76%. Molecules I, II, and III were modeled as visible residues 42–135 and 138–248, 41–133 and 137–249, and 40–135 and 138–249, respectively. Interestingly, the electron density maps of the N-terminal region (Met1–His39) were invisible in all three molecules, indicating that this region is highly disordered (hereinafter referred to as disordered N-terminal region, DNR). The monomeric structure of *Cg*1261 showed an overall fold similar to that of *Cg*LOG, and these two proteins have an amino acid identity of 26% (Fig. 1a)^7^. The *Cg*1261 monomer forms a Rossmann α/β structure and is composed of eight α-helices and seven parallel β-strands (Fig. 1b). The root-mean-square deviation (RMSD) values among these three monomeric structures were under 0.4, indicating that the three monomers have quite similar structures. Molecules I and II form a dimeric structure, which is similar to the dimeric structure of *Cg*LOG (Fig. 1c). The dimerization is mainly mediated by interactions of hydrophobic patches located at the α1, α5, and α6 helices, and the polar interactions Lys156–Glu181 and Asp46–Lys161 additionally aid the dimerization. Based on the PISA software^12^ computation, the buried interface area was 2,288 Å per monomer, and the percentage of participating residues was 28.5%.

### Hexameric structure of *Cg*1261

**Figure 2:**
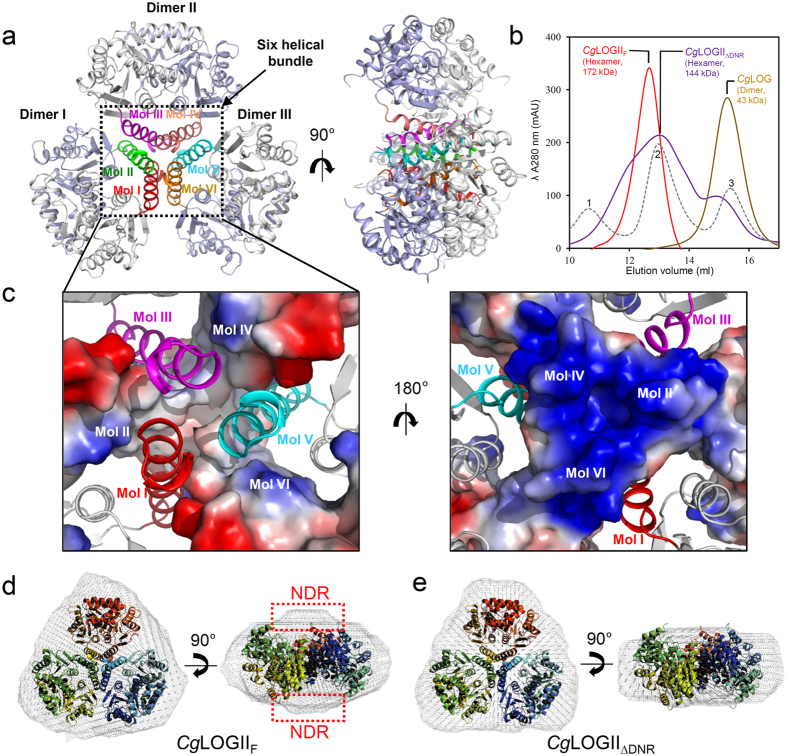
Hexameric structure of *Cg*LOGII. (**a**) Hexameric structure of *Cg*LOGII. The hexameric structure of *Cg*LOGII is presented as a cartoon diagram. The additional α-helices from six different monomers are distinguished with different colors. The right figure is the left figure rotated horizontally by 90°. (**b**) Size-exclusion chromatography of *Cg*LOGII. The full-length (*Cg*LOGII_F_) and the DNR-truncated form (*Cg*LOGII_ΔDNR_) of *Cg*LOGII are eluted as a hexameric form, and *Cg*LOG is as a dimeric form. 1, 2, and 3 indicate standard samples of Ferritin (440 kDa), Aldolase (158 kDa), and Ovalbumin (44 kDa), respectively. (**c**) Hexamerization mode of *Cg*LOGII. Three monomers are presented as an electrostatic potential surface model and the other three monomers are as a cartoon diagram. The right figure is the left figure rotated horizontally by 90°. (**d**) (e) SAXS 3-D reconstructed models of *Cg*LOGII_F_ (**d**) and *Cg*LOGII_ΔDNR_ (**e**), respectively. The right figures are the left figure rotated vertically by 90°. The DNR region in *Cg*LOGII_F_ is indicated with a red-colored dotted rectangle.

Although the dimeric structure of *Cg*1261 resembles that of the *Cg*LOG dimer, *Cg*1261 forms a hexameric structure. The *C*222_1_ crystallographic symmetry generated a hexameric structure (Fig. 2a), which was consistent with the size-exclusion chromatography results (Fig. 2b). The hexameric structure was formed by oligomerization of three dimeric structures, and the overall shape of the hexamer looked like a trillium flower, in which each petal was made up of a twisted dimer. The α1 helices of six monomers mainly contribute to hexamerization of *Cg*1261. The helices form an antiparallel helix bundle through a mixture of hydrogen bonds, salt bridges, and hydrophobic interactions (Fig. 2c). A detailed explanation of the α1 helix and the structural comparison with *Cg*LOG will be provided later. Using the PISA software^12^, the buried surface of the six helices was computed to be 2,409 Å. In order to confirm the oligomeric status of *Cg*1261 in solution and to investigate the relative position of DNR in the *Cg*1261 hexamer, we performed small-angle X-ray scattering (SAXS) analysis in solution using full-length (*Cg*1261_F_) and DNR-truncated (*Cg*1261_ΔDNR_) *Cg*1261 proteins (Supplementary Fig. S1). Consistent with the crystallographic and size-exclusion chromatography results, the SAXS results suggested that *Cg*1261 existed as a hexamer in solution. Because both *Cg*1261_F_ and *Cg*1261_ΔDNR_ proteins formed a hexamer in solution, we suspected that DNR is not a main contributor in hexamerization (Fig. 2b and Supplementary Fig. S1). We then performed three-dimensional (3D) reconstructed structure modeling using the SAXS data. Our *Cg*1261 crystal structure fit well to a 3D reconstructed model of *Cg*1261_ΔDNR_. Compared with the SAXS model of *Cg*1261_ΔDNR_, that of *Cg*1261_F_ showed a bulged structure at both sides of the *Cg*1261_ΔDNR_ model (Fig. 2d,e). These results suggest that DNRs from three monomers are located on the top of the hexameric disc and those from the other three monomers are on the bottom of the disc (Fig. 2d,e).

### Structural comparison of *Cg*1261 with LOG homologs

**Figure 3:**
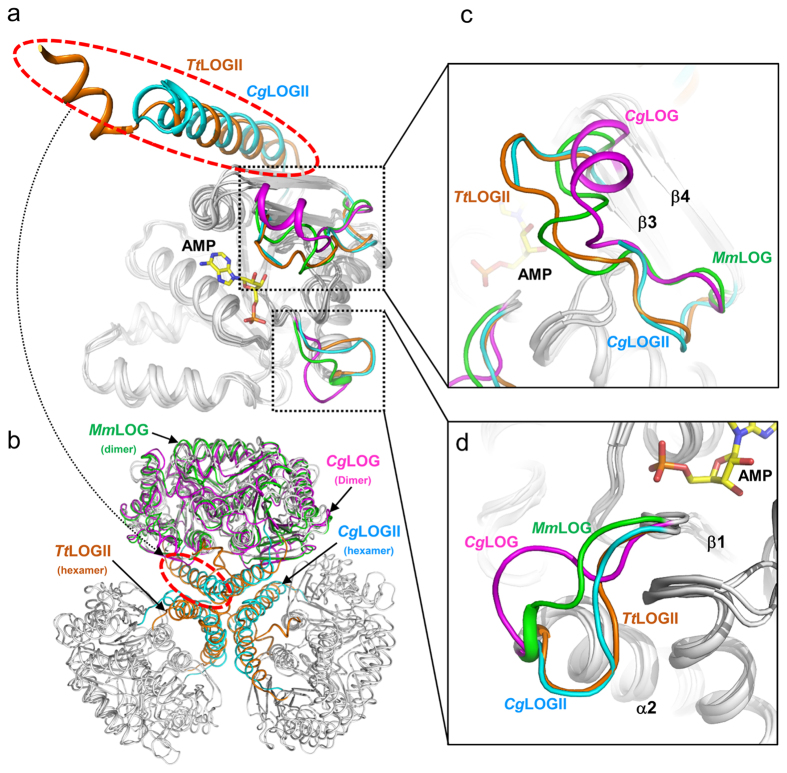
Structural comparison of *Cg*LOGII with other LOGs. (**a**) Structural comparison of the LOG monomers. Four LOGs such as *Cg*LOGII, *Tt*LOGII, *Cg*LOG, and *Mm*LOG are superposed (PDB code 5QW3, 1WEK, 5ITS, and 3SBX, respectively). The additional α-helices from *Cg*LOGII and *Tt*LOGII are indicated with a red-colored circle and distinguished with colors of cyan and orange, respectively. Two structurally variable regions, connecting loops of β1-α2 and β3-β4, are also distinguished with different colors. (**b**) The hexameric forms of *Cg*LOGII and *Tt*LOGII are superposed with the dimeric forms of *Cg*LOG and *Mm*LOG. The additional α-helices from *Cg*LOGII and *Tt*LOGII are indicated with a red-colored circle and distinguished with colors of cyan and orange, respectively. (**c**) Connecting loops of β3-β4 in LOGs. Four LOGs such as *Cg*LOGII, *Tt*LOGII, *Cg*LOG, and *Mm*LOG are superposed, and the connecting loops of these LOGs are distinguished with colors of cyan, orange, magenta, and green, respectively. The bound AMP in *Mm*LOG is presented as a stick model with a yellow color. The secondary structure elements of β3 and β4 are labeled. (**d**) Connecting loop of β1-α2 in LOGs. Four LOGs such as *Cg*LOGII, *Tt*LOGII, *Cg*LOG, and *Mm*LOG are superposed, and the connecting loops of these LOGs are distinguished with the same color scheme as in (C). The secondary structure elements of β1 and α2 are labeled.

Because *Cg*1261 is an uncharacterized LOG isoform, comparisons between *Cg*1261 and other LOGs may elucidate the functional and structural implications of *Cg*1261. First, we compared the structure of *Cg*1261 with that of *Cg*LOG. When we superposed these two structures, the monomeric structures of these two proteins showed a similar overall fold between each other (Fig. 3a, Supplementary Fig. S2). The most conspicuous distinction between these proteins was observed at their N-terminal regions (Figs 1a and 3a). Compared with *Cg*LOG, *Cg*1261 contained an extended N-terminal region, which formed an α1 helix (Asp46–Leu64) (Fig. 3a). Since, as described above, the α1 helix is the main contributor to the hexameric architecture of *Cg*1261 (Fig. 3b), the additional helix is a determinant of the oligomeric state of *Cg*1261. The α1 helix is located distally to the active site (Fig. 3a), and we speculated that the addition of the α1 helix and hexamerization of *Cg*1261 would not influence the enzymatic activity of the protein. When we compared the dimeric structures of *Cg*1261 and *Cg*LOG, noticeable differences were also observed (Fig. 3a, Supplementary Fig. S2). First, the β3–β4 loop (Ile131–Leu145) was highly disordered in *Cg*1261, whereas the corresponding region (Thr74–Glu89) of *Cg*LOG formed a one-turn helix (Fig. 3c). Because this region is quite diverse in various LOGs and is located in the vicinity of the ribose ring of the substrate^7^, we suspect that the stabilization mode of the ribose ring may be somewhat different between these two proteins. Second, the β1–α2 loop (Arg78–His83) of *Cg*1261 was flipped 180° compared with the corresponding region (Ala20–Ser25) of *Cg*LOG (Fig. 3d). The region is located distally to the active site, and the difference does not seem to influence the enzyme activity.

The results of Dali analysis^13^ showed that the most structurally similar protein to *Cg*1261 was *Tt*1465 (Table 2). Although the function of *Tt*1465 has not been experimentally defined yet, the protein with a hexameric structure was reported as a potential LDC enzyme^8^. To compare the structure of *Cg*1261 with that of *Tt*1465, we superposed the structures of these two proteins (Supplementary Fig. S2). Interestingly, the hexamerization modes of these two proteins were almost identical to each other. Similar to *Cg*1261, the additional α-helices at the N-terminal regions of six monomers formed a six-helical bundle at the core of the *Tt*1465 hexamer (Fig. 3b). Moreover, the dimeric structure and active site formation of these proteins were also almost identical to each other, indicating that *Cg*1261 and *Tt*1465 might have a similar function. One noticeable difference between these two proteins, observed at the N-terminal region, was that unlike *Cg*1261, which has a DNR with 40 amino acids at the N-terminal region, *Tt*1465 lacked the DNR region (Fig. 1a). Because the DNR region is located distal from the active site, we suspect that the difference is irrelevant to the function of the proteins. The Dali analysis results also showed that the other proteins of high structural similarity to *Cg*1261 were cytokinin-related LOGs from organisms such as *Arabidopsis thaliana, Claviceps purpurea, Mycobacterium marinum*, and *C. glutamicum* (Table 2, Supplementary Fig. S2). Except the existence of the additional α1 helix at the N-terminal region and the hexameric oligomerization by the helix, *Cg*1261 formed a dimeric component with a mode similar to that in known dimer-forming LOGs (Fig. 3b). Moreover, the active site formation of *Cg*1261 was also quite similar to that of LOGs, which will be described later. These structural analysis data imply that the *Cg*1261 and *Tt*1465 hexamers have a function similar to that of known LOG dimers.

### Active site of *Cg*1261

**Figure 4:**
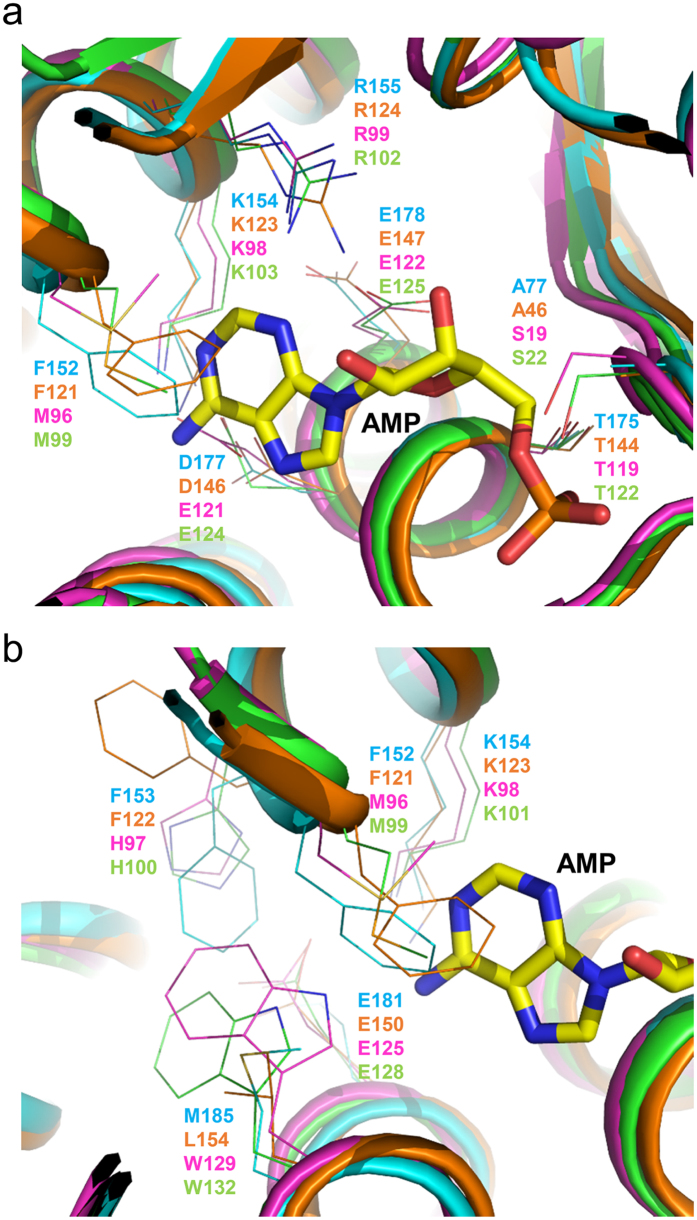
Active site comparison of *Cg*LOGII with homologous proteins. *Cg*LOGII is shown as a cartoon diagram in cyan color scheme. The *Cg*LOG structure is superposed with LOGs such as *Tt*LOGII, *Cg*LOG, and *At*LOG3, and those LOGs are presented as cartoon diagrams in orange, magenta, and green, respectively. The AMP molecule was prepared as in Fig. 3 and shown as a stick model in yellow. Secondary structure elements are labeled. (**a**) AMP binding site. Residues involved in the constitution of the AMP binding site are shown as line models. (**b**) Prenyl-group binding site. Residues involved in the constitution of the AMP binding site are shown as line models.

Superposition of *Cg*1261 with a LOG protein from *M. marinum (Mm*LOG) in complex with AMP (Protein Data Bank code 3SBX) revealed the active site conformation of *Cg*1261. As observed in other LOGs^7^, the active site of *Cg*1261 is located near the PGGxGTxxE motif. The motif has been known to serve as a monophosphate nucleotide-binding structure, and the amino acid residues ^170^PGGFGTLDE^178^ constitute the motif (Fig. 1a). The phosphate moiety was found to be hydrogen-bonded with the main-chain Gly171, Phe173, and Gly174 and the side-chain Thr175 (Fig. 4a). The ribose moiety was mainly stabilized by hydrogen bond interactions between Arg155 and two hydroxyl groups of the ribose moiety (Fig. 4a). To stabilize the adenine moiety, a mixture of hydrophobic and hydrophilic residues, such as Phe152, Lys156, and Asp177, formed the adenine-binding site (Fig. 4a). Two catalytic residues, Arg155 and Glu178, are located near the covalent bond between adenine-N^9^ and ribose-C^1^, which is hydrolyzed by the enzyme. In addition, based on a previous prediction^7^, the Phe152, Phe153, Lys156, Glu181, and Met185 residues seemed to form a prenyl group-binding site (Fig. 4b). Detailed structural comparison of the active site of *Cg*1261 with those of other LOGs and *Tt*1465 revealed that the residues constituting the modified adenine-binding site were somewhat variable, whereas those involved in the stabilization of the phosphoribose moiety were mostly conserved (Fig. 4a,b). The *Cg*1261 and *Tt*1465 hexamers utilize Phe152 and Asp177 to stabilize the adenine moiety, whereas known LOG dimers contain methionine and glutamate residues at the corresponding positions (Fig. 4b). The residues constituting the prenyl group-binding site are more variable in different proteins. Instead of Phe152 and Phe153 present in *Cg*1261, the *At*LOG3 and *Cg*LOG dimers contain methionine and histidine, respectively, at the corresponding positions, whereas *Tt*1465 also has phenylalanine residues at the same positions (Fig. 4b). At the position of Met185 in *Cg*1261, a leucine residue is located in *Tt*1465 and a tryptophan residue is in the *At*LOG3 and *Cg*LOG dimers. However, most importantly, two catalytic residues, Arg155 and Glu178, are located at the same positions in all four proteins, including *Cg*1261. Taken together, these structural observations led us to propose that the hexameric form of LOG-like proteins such as *Cg*1261 and *Tt*1465 might have a function similar to that of the dimeric form of LOGs such as *At*LOG3 and *Cg*LOG. The structural difference at the prenyl group-binding site also suggests that, compared to the dimeric LOGs, the hexameric form of LOG-like proteins may accommodate a modified AMP, with a slightly different prenyl group, as a substrate.

### *Cg*1261 has a LOG function

**Figure 5:**
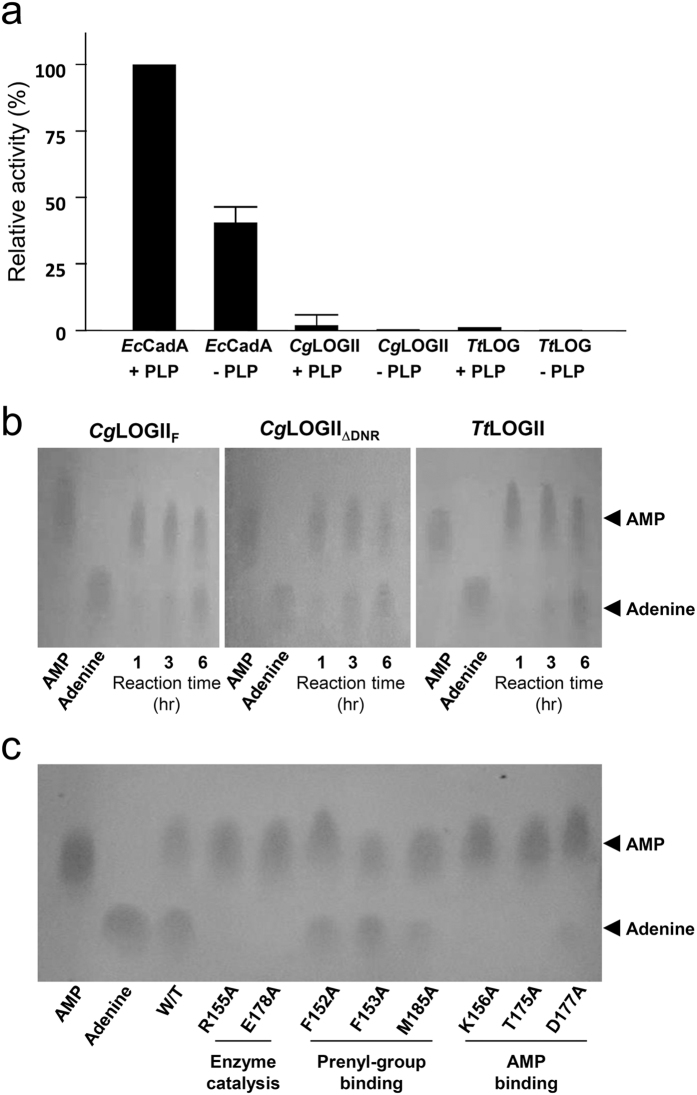
Phosphoribohydrolase activity of *Cg*LOGII. (**a**) Lysine decarboxylase activity assay of *Ec*CadA, *Cg*LOGII, and *Cg*LOG. The lysine decarboxylase activity of *Ec*CadA, *Cg*LOGII, and *Cg*LOG are measured with or without PLP. All experiments are performed in triplicates. (**b**) Phosphoribohydrolase activity of *Cg*LOGII_F_, *Cg*LOGII_ΔDNR_ and *Tt*LOGII. The activities of *Cg*LOGII and *Tt*LOGII were measured at 30 and 65 °C, respectively. The AMP and adenine standards are indicated at the right side of the figure. (**c**) Site-directed mutagenesis experiments of *Cg*LOGII. The residues involved in the enzyme catalysis, prenyl-group binding site and the AMP binding were replaced by alanine. The reaction mixture containing each mutant was incubated for 6 hours.

To confirm that the *Cg*1261 and *Tt*1465 hexamers function as LOG proteins, rather than LDCs as suggested by structural observations, we performed LDC and phosphoribohydrolase activity assays using these proteins and *Cg*LOG. As expected, *Cg*1261 and *Tt*1465 exhibited no LDC activity (Fig. 5a), indicating that, contrary to the previous annotation, neither protein is an LDC. However, the phosphoribohydrolase activity assays showed that both *Cg*2612 and *Tt*1465 hydrolyzed AMP into an adenine base and ribose 5-phosphate, and the hydrolase activities tended to increase with the reaction time (Fig. 5b). Although the conversion of AMP to adenine and ribose 5-phosphate requires a long incubation time, the levels of the hydrolyzing activities of these proteins were quite similar to that of *Cg*LOG^7^. In addition, involvement of the suggested residues in enzyme catalysis and substrate binding was also confirmed by site-directed mutagenesis experiments (Fig. 5c). As expected, substitutions of the Arg155, Lys156, Thr175, and Glu178 residues with alanine resulted in a complete loss of phosphoribohydrolase activity, and a Asp177Ala substitution resulted in an almost complete loss of the activity. However, the proteins with their prenyl group-binding residues, such as Phe152, Phe153, and Met185, substituted with alanine still showed the activity because we performed the assay using AMP as a substrate. These results indicate that the hexameric form of LOG-like proteins such as *Cg*2612 and *Tt*1465 has a LOG function with a similar substrate-binding mode. It is worth noting that *Cg*1261_ΔDNR_ showed the same level of phosphoribohydrolase activity as *Cg*1261 (Fig. 5b), indicating that the DNR region is not involved in the enzyme reaction, as we previously suggested based on the structural observations.

**Figure 6:**
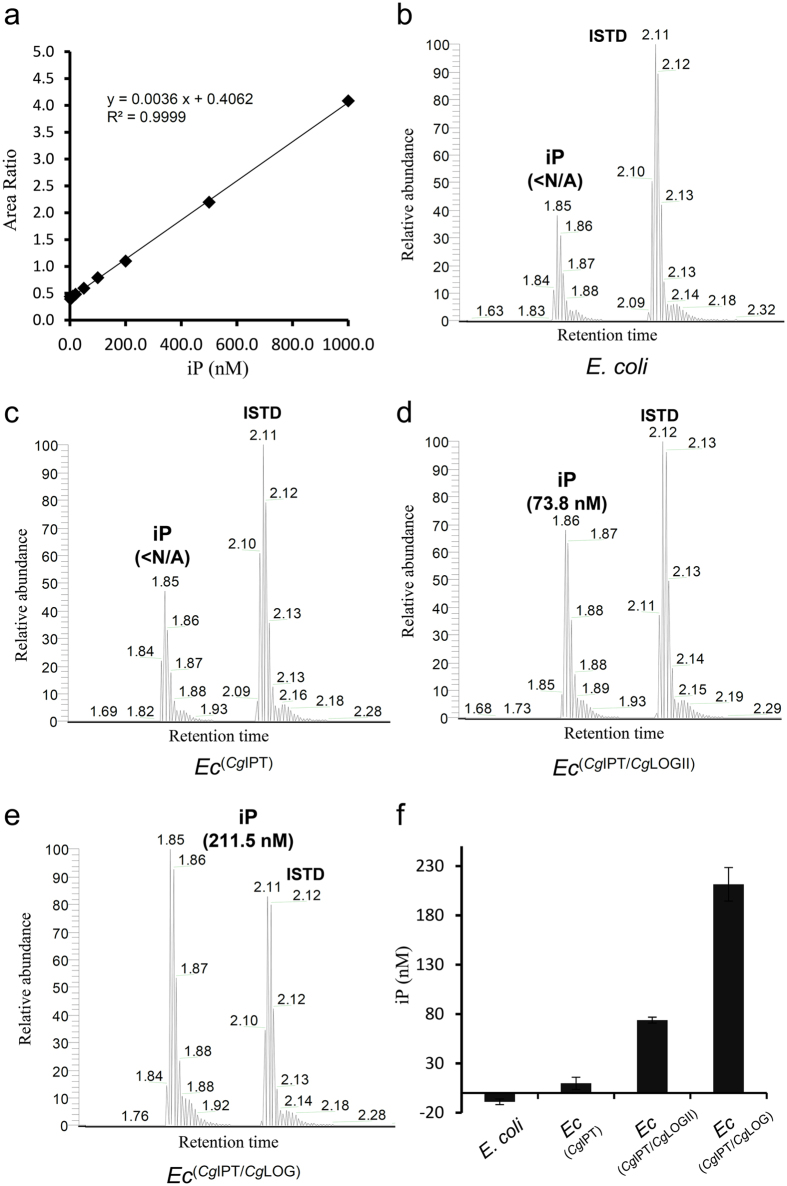
*In vivo* cytokinin production. (**a**) Linear calibration curve of iP standard. Trend line equation, R^2^, and the standard curve range were indicated. (**b**–**e**) The production of cytokinin in *E. coli* strains expressing no extra gene (**b**), *Cg*IPT (**c**), *Cg*IPT/*Cg*LOGII (**d**) and *Cg*IPT/*Cg*LOG (**e**) were selectively monitored using high pressure liquid chromatography-tandem mass spectrometry.<N/A is not available because it deviated from the credible standard curve range. (**f**) Histograms of detected iP concentration from (**b**) to (**e**) were presented with standard deviations.

In cytokinin biosynthesis, the most important enzyme is isopentenyltransferase (IPT), which transfers the prenyl group of dimethylallyl pyrophosphate to adenylate or transfer RNA (tRNA). We have previously expected that *C. glutamicum* may produce cytokinins through a tRNA-mediated pathway and that the IPT (*Cg*IPT) encoded by the *Cg2130* gene is involved in the pathway^7^. To further confirm the LOG function of *Cg*1261, we performed cytokinin (iP) production experiment in *Escherichia coli* and monitored the iP production by a liquid chromatography–tandem mass spectrometry method (Fig. 6a–f). No noticeable iP production was detected in cultures of an *E. coli* strain without a heterologous gene and a strain expressing only the *Cg*IPT-coding gene (*Ec*^*Cg*IPT^) (Fig. 6b,c,f). However, a significant amount of iP was detected in a culture of an *E. coli* strain expressing both the *Cg*IPT- and *Cg*1261-coding genes (*Ec*^*Cg*IPT/*Cg*1261^) (Fig. 6d,f). The results confirm that *Cg*1261 has a cytokinin-activating function. To compare iP production levels between *Cg*1261 and *Cg*LOG, we also monitored the production of iP using an *E. coli* strain expressing the *Cg*LOG-coding gene (*Ec*^*Cg*IPT/*Cg*LOG^) instead of the *Cg*1261-coding gene. The *Ec*^*Cg*IPT/*Cg*LOG^ strain produced 2~3 times more iP than the *Ec*^*Cg*IPT/*Cg*1261^ strain (Fig. 6e,f). The difference in the iP production between *Cg*1261 and *Cg*LOG could be caused by differences in the protein expression levels in *E. coli* and/or by those in amino acid residues at the active site of these two enzymes. Taken together, based on the structural and biochemical observations on *Cg*1261, we propose that hexameric LOG-like proteins such as *Cg*1261 and *Tt*1465 have the same cytokinin-activating function as known dimeric LOG proteins.

### Classification of LOG proteins

**Figure 7:**
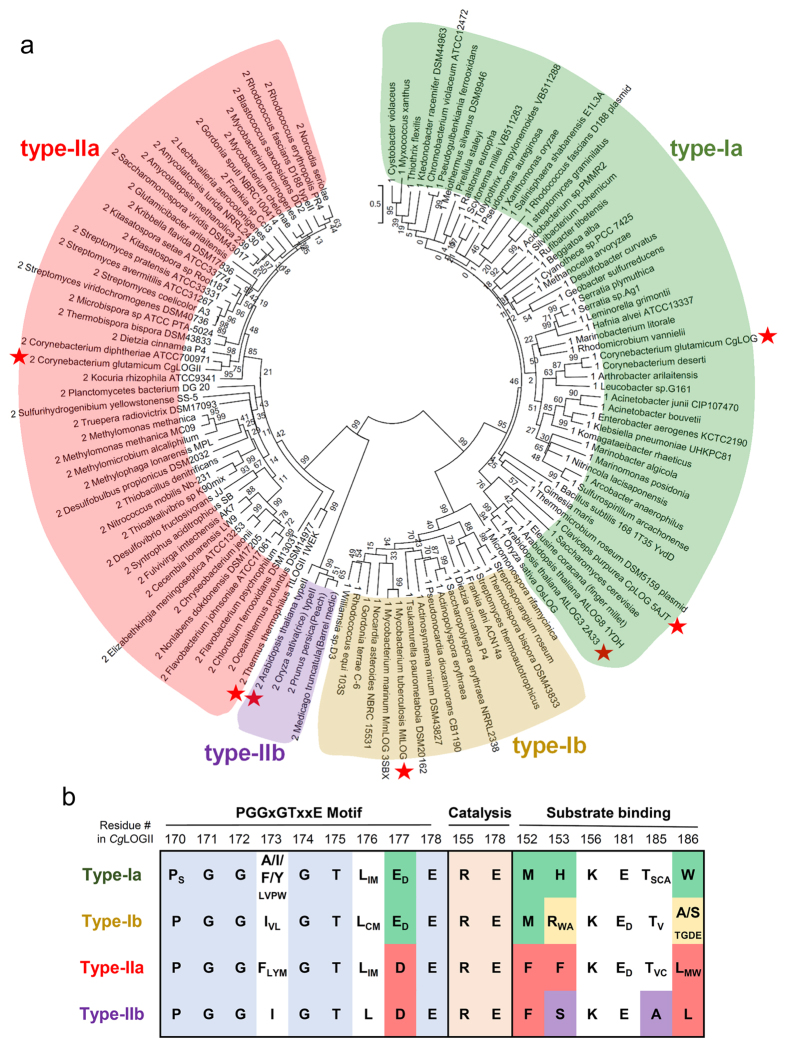
Phylogenetic tree and comparative analysis of LOG proteins. (**a**) Unrooted Maximum Likelihood tree of LOG proteins. The phylogenetic tree was drawn as a circle model. Bootstrap values are shown at each node as percentage of 100 replicates. Four subgroups are labeled as different color schemes. Typical LOGs referred in the manuscript are indicated by stars. (**b**) Amino acid sequence alignment of key residues in LOGs. The key residues involved in the PGGxGTxxE motif, enzyme catalysis, and substrate binding in four subgroups of LOGs are aligned.

Our study revealed that the proteins such as *Cg*1261 and *Tt*1465 are not LDCs but rather a novel type of cytokinin-activating proteins. Because, compared with known LOGs, this type of LOG family proteins shows differences in the oligomeric state and in the residues at the prenyl group-binding site, classification of LOG proteins is required. Thus, we analyzed 123 LOG-like proteins from various phylogenetically diverse organisms found in multifarious habitats and constructed a maximum-likelihood phylogenetic tree (Supplementary Fig. S3). The majority of the sequences were well matched with the LOG motif based on our multiple alignment data. As expected, the LOG-like proteins were categorized into two clusters with high bootstrap values (Fig. 7a). One cluster contained dimeric LOGs, including *Cg*LOG and *At*LOG3, and the other contained hexameric LOGs, including *Cg*1261 and *Tt*1465. Therefore, we classified the cluster containing the dimeric LOGs as type I LOGs and that containing the hexameric LOGs as type II LOGs. Based on this classification, we refer to the *Cg*1261 and *Tt*1465 proteins as *Cg*LOGII and *Tt*LOGII, respectively. Interestingly, the phylogenetic analysis suggested that the type I LOGs could be divided into two subgroups, type Ia and type Ib (Fig. 7a). Type Ia included dimeric LOGs from most organisms such as *A. thaliana, C. purpurea*, and *C. glutamicum*. Type Ib included dimeric LOGs from the *Actinomycetales*, including mammalian pathogens such as *Mycobacterium tuberculosis, Nocardia asteroides*, and *Rhodococcus equi*. The phylogenetic analysis also suggested that the type II LOGs could be divided into two subgroups, type IIa and type IIb (Fig. 7a). Type IIa included hexameric LOGs from most organisms, except higher plants, which could be categorized as type IIb.

These four subgroups showed their own synapomorphies in the residues at the active site (Fig. 7b). The residues involved in the enzyme catalysis in *Cg*LOGII, Arg155 and Glu178, were found to be completely conserved throughout all LOG subgroups (Fig. 7b), indicating that the function of all LOG family enzymes involves the same catalytic mechanism. The PGGxGTxxE motif is also highly conserved in all subgroups (Fig. 7b). One noticeable difference was found in position 177, which is occupied in *Cg*LOGII by aspartate, a residue involved in the binding of the adenine ring moiety. The type II LOGs contained aspartate residues at the corresponding position, while the majority of type I LOGs contained glutamate residues (Fig. 7b). However, the residues involved in substrate binding were somewhat variable among the different types and subgroups. In particular, different residues were found in the positions occupied by Phe152, Phe153, and Met186 in *Cg*LOGII (Fig. 7b). These residues are all involved in the constitution of the prenyl group-binding site, and we suspect that LOGs from the different types and subgroups may produce cytokinin compounds with somewhat different modifications at the position of the prenyl group.

## Discussion

None of the type II LOG proteins has been functionally characterized so far, and our structural and biochemical studies of *Cg*LOGII revealed that this new type of LOG proteins exists in a variety of organisms, in addition to known type I LOGs. LOGs have been known to function in plants and plant-interacting organisms as phytohormone producers. Because *C. glutamicum* is a soil bacterium, it can be speculated that the microorganism also utilizes cytokinins produced by its LOGs to interact with plants. However, the fact that a variety of microorganisms, including mammalian pathogens, have LOG-coding genes raises two hypotheses about the function of LOGs. First, LOGs in some organisms may be involved in the production of different forms of cytokinins, not phytohormone cytokinins. Second, cytokinins may have cellular functions other than those of phytohormones in a variety of microorganisms. Thus, investigations on cellular functions of cytokinins and their analogs in bacterial cells are required.

When we analyzed the operons containing type II LOG-coding genes and their neighboring genes, genes encoding for succinyl-diaminopimelate desuccinylase (DapE), dihydropteroate synthase, glucosyl-3-phosphoglycerate synthase, and a methyltransferase were found to be located close to the type II LOG-coding genes. It is interesting that DapE, an enzyme in the lysine biosynthetic pathway, is located close to the type II LOG-coding genes, which seems to be one of the reasons for misannotation of the LOG-like protein as LDC. More interestingly, the phytopathogen *Rhodococcus fascians* has genes encoding both types of LOGs, which are located in tandem, indicating that both types of LOGs have similar cellular functions. Most importantly, *A. thaliana* contains the coding gene (*At2g50575*) for a type IIb LOG, as well as those for known dimeric forms of LOGs. Further investigations are crucial to reveal whether the type IIb LOG protein is another enzyme for the production of phytohormones.

Molybdenum cofactor carrier proteins (MCPs), known to transfer a molybdenum cofactor to molybdenum enzymes^14^,^15^,^16^, are considered LOG-like proteins because of their structural similarity to LOGs. However, the active site conformation of MCPs is completely different from that of LOG proteins, and the residues involved in the enzyme catalysis and constitution of the PGGxGTxxE motif are not conserved in MCPs. Especially, the arginine residue involved in the LOG enzyme catalysis is replaced by alanine in MCPs. Based on these differences between MCPs and LOG proteins, we suggest that MCPs are excluded from the LOG family of proteins.

## Materials and Methods

### Cloning, expression, and purification

The genes corresponding for *Cg*1261 (*Cg*LOGII) from *Corynebacterium glutamicum* ATCC 13032 was amplified from genomic DNA of *C. glutamicum* by polymerase chain reaction (PCR) with primers: forward, 5-GCGC**CATATG**GCTCCTAAACAAACTCCCAGC-3, and reverse, 5-GCGC**CTCGAG**ATTGTGGCGACGCGCTACGTCC-3. The PCR product was then subcloned using restriction endonucleases *Nde*I and *Xho*I into pET30a vector (Merck Millipore) with 6xHis tag at the C-terminus. The resulting expression vectors pET30a: *Cg*LOGII was transformed into *E. coli* BL21 (DE3) strain and the cell were grown on LB medium containing 100 mgl^−1^ kanamycin at 37 °C to OD600 of 0.6. The cell was induced by adding 1.0 mM Isopropyl 1-thio-β-D-galactopyranoside (IPTG) for 20 h at 18 °C and harvested by centrifugation at 4000 rpm for 20 minute. Harvested cells was resuspended in ice-cold lysis buffer (40 mM Tris-HCl, pH 8.0) and disrupted by ultrasonication. The cell debris was removed by centrifugation at 11,000 × g for 1 h, and the supernatant was loaded on to Ni-NTA agarose column (QIAGEN). After washing with lysis buffer containing 18 mM imidazole, the bound proteins were eluted with 300 mM imidazole in lysis buffer. Further purification was carried out by applying the HiTrap Q ion exchange chromatography and size exclusion chromatography using Sephacryl-300 (320 ml, GE Healthcare). The purified proteins were concentrated to 32 mg ml^−1^ in 40 mM Tris–HCl, pH 8.0, and stored at −80 °C for crystallization trials. Site-directed mutagenesis experiments were performed using the QuikChange site-directed mutagenesis kit (Stratagene). The production and purification of the *Cg*LOGII mutants were carried out by the same procedures as described for the wild-type protein. *Tt*1465 from *Thermus thermophilus (Tt*LOGII) was prepared by the procedure similar to *Cg*LOGII. CgLOG and CadA from *E. coli (Ec*CadA) were prepared as described in ref. 7.

### Crystallization, Data collection, and Structure determination

Crystallization of the purified proteins were initially performed by the hanging-drop vapor-diffusion method at 20 °C using commercially available sparse-matrix screens from Hampton Research and Emerald BioSystems. Each experiment consisted of mixing 1.0 μl protein solution with 1.0 μl reservoir solution and then equilibrating it against 0.5 ml of the reservoir solution The *Cg*LOGII crystals were observed from several crystallization screening conditions. After several optimization steps using the hanging-drop vapor-diffusion method, the best-quality crystals appeared in 11 day using a reservoir solution consisting of 0.2 M Lithium chloride and 26% PEG 3350 and reached maximal dimensions of approximately 0.6 × 0.5 × 0.1 mm. For the cryo-protection of the crystals, glycerol of 30% glycerol in reservoir solution was used. Data were collected at 100 K at 7 A beamline of the Pohang Accelerator Laboratory (Pohang, Korea) using a Quantum 270 CCD detector (San Diego, CA, USA). The data were then indexed, integrated, and scaled using the HKL2000 program^17^. Crystals of *Cg*LOGII belonged to the C-centered orthorhombic space group *C*222_1_, with unit cell constants of *a* = 98.7 Å, *b* = 173.6, Å *c* = 79.9 Å. Assuming three molecules of *Cg*LOGII per asymmetric unit, the crystal volume per unit of protein mass was approximately 1.99 Å^3^·Da^−1^, which corresponds to a solvent content of approximately 38.38%^18^. To solve the structure of *Cg*LOGII, phasing was carried out by molecular replacement method. The molecular replacement was performed by *MOLREP*^19^ using the structure of possible lysine decarboxylase *Tt*1465 from *T. thermophilus* HB8 (PDB code 1WEK) approaching 49% amino acid identity as a search model. The model building was performed using the program *WinCoot*^20^ and the refinement was performed with *REFMAC5*^21^. The data statistics are summarized in Table 1. The refined model of *Cg*LOGII was deposited in the Protein Data Bank (PDB code 5WQ3).

### Size-exclusion chromatographic analysis

To investigate the oligomerization of *Cg*LOGII_F_ and *Cg*LOGII_ΔDNR_, analytical size-exclusion chromatography was performed using a Superdex 200 10/300 column (GE Healthcare) at NaCl concentrations of 150 mM. Protein samples of 1 mL with concentration of 3 mg/ml were analyzed. The molecular weights of the eluted samples were calculated based on the calibration curve of standard samples.

### Solution SAXS measurements

Small-angle X-ray scattering (SAXS) measurements were carried out using the 4 C SAXS II beamline of the Pohang Accelerator Laboratory (Pohang, Korea) with 3 GeV power. A light source from an In-vacuum Undulator 20 (IVU20: 1.4 m length, 20 mm period) of the Pohang Light Source II storage ring was focused with a vertical focusing toroidal mirror coated with rhodium and monochromatized with a Si (111) double crystal monochromator (DCM), yielding an X-ray beam wavelength of 0.734 Å. The X-ray beam size at the sample stage was 0.1 (V) × 0.3 (H) mm^2^. A two-dimensional (2D) charge-coupled detector (Mar USA, Inc.) was employed. A sample-to-detector distance (SDD) of 4.00 m and 1.00 m for SAXS were used. The magnitude of scattering vector, *q* = (4*π/λ*) sin*θ*, was 0.1 nm^−1^ < *q* < 6.50 nm^−1^, where 2*θ* is the scattering angle and *λ* is the wavelength of the X-ray beam source. The scattering angle was calibrated with polystyrene-b-polyethylene-b-polybutadiene-b-polystyrene (SEBS) block copolymer standard. We used quartz capillary with an outside diameter of 1.5 mm and wall thickness of 0.01 mm, as solution sample cells. All scattering measurements were carried out at 4 °C by using a FP50-HL refrigerated circulator (JULABO, Germany). The SAXS data were collected in six successive frames of 0.1 min each to monitor radiation damage. Measurements of LOG protein solutions were carried out over a small concentration range 0.5 ~4.5 mg/mL. Each 2D SAXS pattern was radial averaged from the beam center and normalized to the transmitted X-ray beam intensity, which was monitored with a scintillation counter placed behind the sample. The scattering of specific buffer solutions were used as the experimental background. The R_g,G_ (radius of gyration) values were estimated from the scattering data using Guinier analysis^22^. The molecular mass (MM) was calculated from the scattering curve based on the Q_R_ method^23^. The pair distance distribution p(r) function was obtained through the indirect Fourier transform method using the program GNOM^24^.

### Construction of 3D structural models

To reconstruct the molecular shapes, the ab initio shape determination program DAMMIF^25^ was used. For each model reconstruction, five independent models were selected, and the averaged aligned using the program DAMAVER^26^. The SAXS curves were calculated from the atomic models using the program CRYSOL^27^. For comparison of the overall shapes and dimensions, the ribbon diagrams of the atomic crystal models were superimposed onto the reconstructed dummy atom models using the program SUPCOMB^28^.

### Lysine decarboxylase activity assay

Lysine decarboxylase activity assay was performed as described in ref. 7. The activity of LDC was determined by measuring residual concentration of _L_-lysine using lysine oxidase and peroxidase. After LDC reaction, lysine oxidase converts remaining lysine into 6-amino-2-oxohexanoate, NH_3_, and H_2_O_2_ and then the hydrogen peroxide is reduced by peroxidase with 2,2′-azino-bis(3-ethylbenzothiazoline-6-sulphonic acid) (ABTS). The oxidized ABTS is detected by spectrophotometric method in absorbance at 412 nm. The assay was performed at 30 °C in a total volume 200 μl, containing 100 mM potassium phosphate, pH 6.0, 0.1 M _L_-lysine, 0.2 mM pyridoxal-5-phosphate, and 25 μg of purified enzymes. The reaction was stopped by heating the reaction mixture at 100 °C for 5 min. After centrifugation at 13,500 × *g* for 1 min, 2X reaction solution that contains 0.1 unit ml^−1^ lysine oxidase and 1 unit ml^−1^ peroxidase in potassium phosphate buffer is added to the reaction mixture.

### Phosphoribohydrolase activity assay

Phosphoribohydrolase activity assay was performed as described in^7^. The activity was determined by detecting adenine ring compounds separated by thin layer chromatography (TLC) method. Enzyme reactions were carried out in the mixture of 20 mM AMP, 36 mM Tris-HCl, pH 8.0, and 23 μM purified enzymes at 30/65 °C and then the reactions were stopped by heating the mixture at 95 °C for 1.5 min. The reaction mixtures were then dotted on PEI-cellulose-F plastic TLC sheet (Merck Millipore). The mobile phase was 1 M sodium chloride. After development in the TLC chamber, the sheet was dried completely. Adenine ring-including compounds were detected by UV lamp (290 nm).

### HPLC-MS/MS analysis

*E. coli* BL21 (DE3) strains containing cytokinin synthesis genes were grown on LB medium at 37 °C to OD600 of 0.6. After induction with IPTG, the cells were grown for 12 h at 18 °C and harvested by centrifugation at 4000 rpm for 20 minute. 2 g of wet cells were resuspended in ice-cold extracted in Bieleski buffer (60% methanol, 25% CHCl3, 10% HCOOH and 5% H2O)^29^ of 15 ml and disrupted by ultrasonication. The cell debris was removed by centrifugation at 11,000 × g for 1 h, and 2 μL aliquot of the supernatant was directly injected into the chromatographic system. Prepared samples were analyzed with a reversed-phase Kinetex XB-C18 column (2.1 × 100 mm, 2.6 μm particle size; Phenomenex, USA) in the Nexera XR system (Shimadzu, Japen). The mobile phase for the HPLC system was solvent A and B; (A) 0.1% formic acid in water and (B) 0.1% formic acid in acetonitrile. The HPLC system was interfaced to a TSQ vantage triple quadrupole mass spectrometer equipped with Xcalibur version 1.1.1 (Thermo, Waltham, USA) operating selected reaction monitoring (SRM) Turbo Ion spray mode in the positive ion as the following m/z transitions: 204 → 136 for iP; 277 → 175 for internal standard (ISTD), a chlorpropamide. To avoid contamination by particles, the mobile phase was filtered via a 0.45 μm filter device (PEEK, Germany) before use. Quantifications of iP were carried out by the calibration curve of standard iP (Sigma-aldrich) ranging 1 to 20000 nM. After comparison between specific amount and calculated amount of iP standards from the curve, quantities in calibration range under 20 nM that showed over 10% of the standard differences were discarded. All experiments are performed in triplicates.

### Phylogenetic tree analysis

Iterative searching for LOG-like proteins was performed by Basic Local Alignment Search Tool (BLAST) in National Center for Biotechnology Information (NCBI) server using position-specific iterated BLAST (PSI-BLAST) method^30^, and also selected using protein sequence and name in BlastKOALA (KEGG Orthology And Links Annotation, http://www.kegg.jp/blastkoala), and the Uniprot protein database (http:// www.uniprot.org/). Multiple alignment was performed by *Clustal omega*^31^. Evolutionary analyses were conducted in MEGA7^32^. The evolutionary history was inferred by using the Maximum Likelihood method based on the Le_Gascuel_2008 model^33^. The tree with the highest log likelihood (−25391.3439) is shown. The percentage of trees in which the associated taxa clustered together is shown next to the branches. Initial tree(s) for the heuristic search were obtained automatically by applying Neighbor-Join and BioNJ algorithms to a matrix of pairwise distances estimated using a JTT model, and then selecting the topology with superior log likelihood value. A discrete Gamma distribution was used to model evolutionary rate differences among sites (5 categories (+G, parameter = 1.8372)). The rate variation model allowed for some sites to be evolutionarily invariable ([+I], 2.6471% sites). The tree is drawn to scale, with branch lengths measured in the number of substitutions per site. The analysis involved 123 amino acid sequences. All positions with less than 95% site coverage were eliminated. That is, fewer than 5% alignment gaps, missing data, and ambiguous bases were allowed at any position. There were a total of 170 positions in the final dataset.

## Additional Information

**How to cite this article**: Seo, H. and Kim, K.-J. Structural basis for a novel type of cytokinin-activating protein. *Sci. Rep.* **7**, 45985; doi: 10.1038/srep45985 (2017).

**Publisher's note:** Springer Nature remains neutral with regard to jurisdictional claims in published maps and institutional affiliations.

## Supplementary Material

Supplementary Information

## Figures and Tables

**Table 1:** Data collection and refinement statistics.

*Cg* LOGII	
**Data collection**	
Space group	*C*222_1_
Cell dimensions	
*a, b, c* (Å)	98.7, 173.6, 79.9
*α, β, γ* (°)	90.0, 90.0, 90.0
Resolution (Å)	86.82-1.95 (1.98-1.95)
*R*_sym_ or *R*_merge_	7.9 (30.8)
*I*/s*I*	39.0 (4.5)
Completeness (%)	95.5 (92.1)
Redundancy	8.5 (4.7)
**Refinement**	
Resolution (Å)	50.00−1.95
No. reflections	45666
*R*_work_ / *R*_free_	17.7/22.7
No. atoms	5281
Protein	4992
Ligand/Water	289
*B*-factors	33.0
Protein	32.7
Ligand/Water	38.1
R.m.s. deviations	
Bond lengths (Å)	0.018
Bond angles (°)	1.830
Resolution (Å)	50.00−1.95

^a^The numbers in parentheses are statistics from the highest resolution shell.

^b^*R*sym = Σ |Iobs−Iavg|/Iobs, where Iobs is the observed intensity of individual reflection and Iavg is average over symmetry equivalents.

^c^*R*work = Σ ||Fo|−|Fc||/Σ |Fo|, where |Fo| and |Fc| are the observed and calculated structure factor amplitudes, respectively. *R*free was calculated with 5% of the data.

**Table 2:** Structural homologues of *Cg*LOGII.

Protein name	PDB code	Z-score with *Cg*LOGII	rmsd (Å)	Sequence Identity (%)	Oligomer	Classification
*Cg*LOGII (*Cg*1261)	5WQ3	N/A	N/A	100	hexamer	Type-IIa
*Tt*LOGII (*Tt*1465)	1WEK	30.8	1.8	49	hexamer	Type-IIa
*Ms*LOG	3QUA	24.2	1.6	26	dimer	Type-Ib
*Mm*LOG	3SBX	23.7	1.6	26	dimer	Type-Ib
*Cg*LOG	5ITS	23.3	1.9	26	dimer	Type-Ia
*At*LOG3	2A33	22.7	1.8	28	dimer	Type-Ia
*At*LOG8	1YDH	22.1	2.0	23	dimer	Type-Ia
*Cp*LOG	5AJT	21.2	2.2	24	dimer	Type-Ia
