# Supplementary material for: Structural basis for a novel type of cytokinin-activating protein
Source: Sci Rep. 2017 Apr 4;7:45985. doi: 10.1038/srep45985 (PMC5379747; doi:10.1038/srep45985)
Supplement: Supplementary Information [file srep45985-s1.pdf]

Structural basis for a novel type of cytokinin-activating protein

Hogyun Seo and Kyung-Jin Kim

Supplementary Information

This Supplementary Information contains 3 Supplementary Figures.

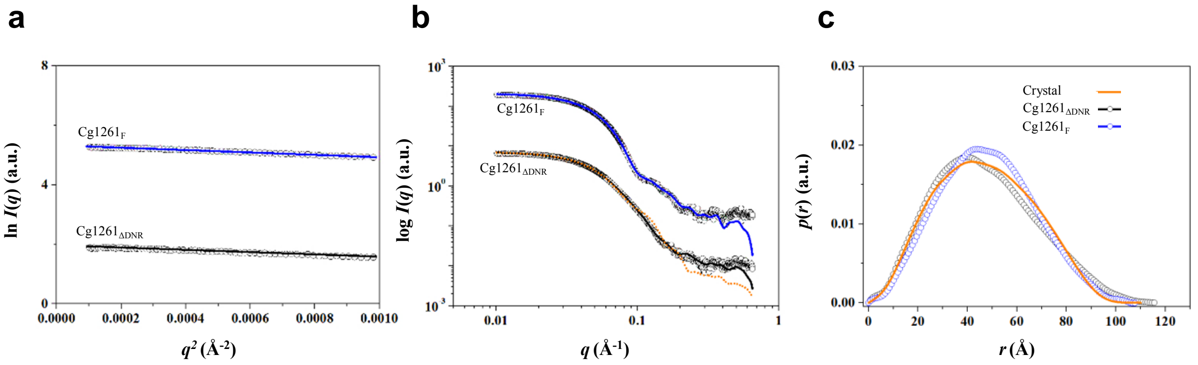

d

Sample	$R_{g,G}$ [a] (Å)	$R_{g,p(r)}$ [b] (Å)	D_{max} [c] (Å)	$MM_{calculated}$ [d] (kDa)	MM_{SAXS} [e] (kDa)	Conformation
Crystal	36.07 ± 0.01	36.02 ± 0.01	110	146	-	hexamer
$Cg1261_{\Delta DNR}$	35.63 ± 0.63	36.35 ± 0.34	116	146	123	hexamer
$Cg1261_F$	35.82 ± 0.49	36.56 ± 0.15	108	172	181	hexamer

[a] $R_{g,G}$ (radius of gyration) was obtained from the scattering data by the Guinier analysis.
[b] $R_{g,p(r)}$ (radius of gyration) was obtained from the p(r) function by the program GNOM.
[c] D_{max} (maximum dimension) was obtained from the p(r) function by the program GNOM.
[d] $MM_{calculated}$ (molecular mass) was obtained from the amino acid sequence of protein.
[e] MM_{SAXS} (molecular mass) was estimated from a BSA standard protein and the scattering curve based on the Q_R method.

Supplementary Figure S1. SAXS analysis of the *Cg*LOGII proteins in aqueous solution. (a) Guinier plot of the X-ray scattering profiles of the *Cg*LOGII proteins. (b) X-ray scattering profiles of the *Cg*LOGII proteins. The open symbols are experimental data and the solid line is the X-ray scattering profiles obtained from the dummy atoms models by the program DAMMIF. The dashed orange line is theoretical SAXS curve calculated from the dimeric crystal structure of *Cg*LOGII using the program CRYSOL. Discrepancy (χ^2) between the experimental and theoretical curve is 0.348. (c) The pair distance distribution $p(r)$ functions for the *Cg*LOGII proteins in solution, based on an analysis of the experimental SAXS data using the program GNOM. (d) Structural parameters obtained from the SAXS data of the *Cg*LOGII proteins.

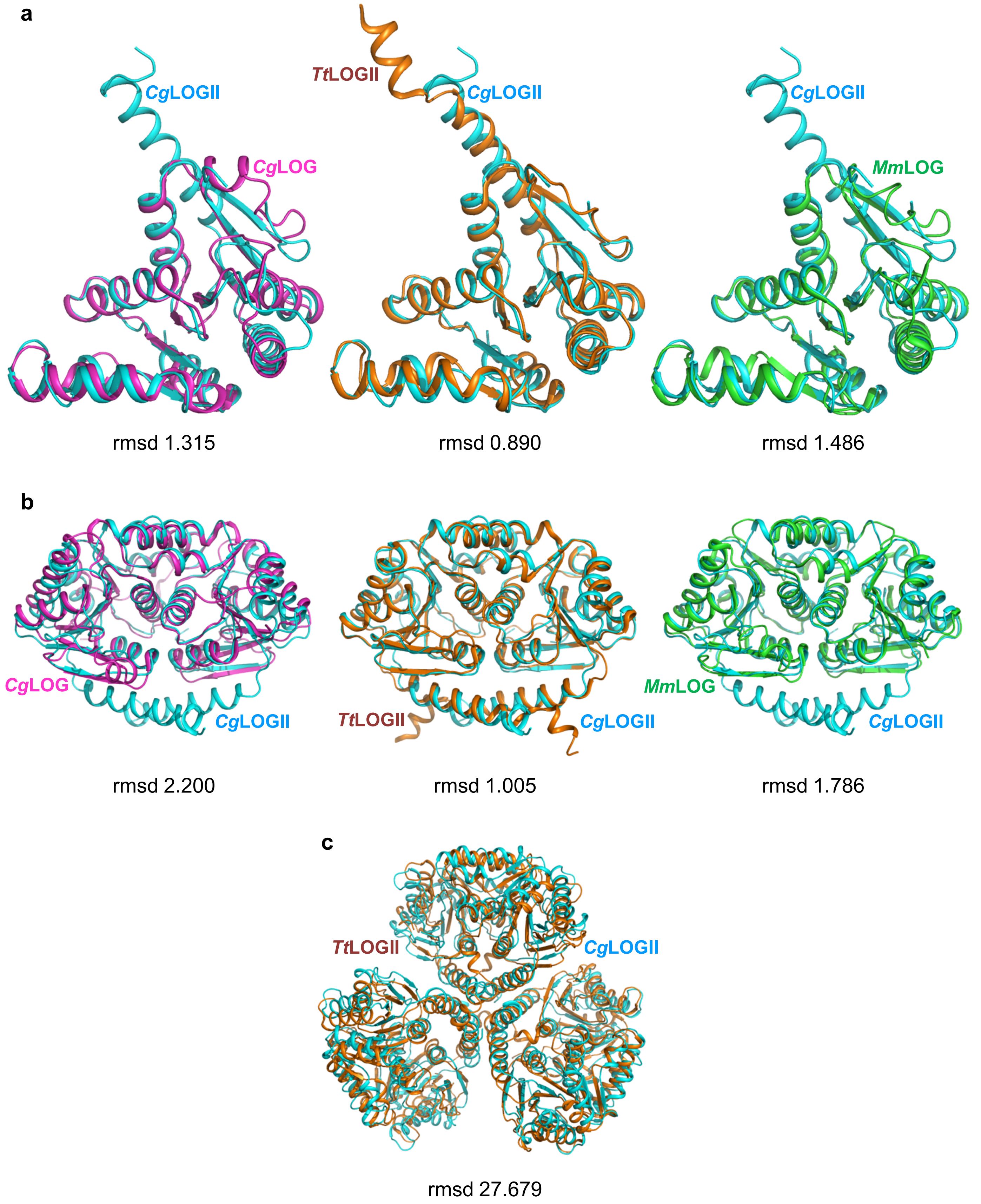

Supplementary Figure S2. The individual superposition of *Cg*LOGII with LOG homologs (a)-(c): superposition of monomers (a), dimers (b), and hexamers (c). All of the structures are displayed as cartoon diagrams with different color schemes as in Fig. 3 and labeled. Each rmsd values are indicated below.

Uniprot (Ncbi) Accession Code	Gene name or Locus name	Amino acids	Assignment
Q8L8B8	***At2g37210***	215	1_Arabidopsis_thaliana_AtLOG3_2A33
Q8NN34	***Cg2612/Cgl2379***	195	1_Corynebacterium_glutamicum_CgLOG
P48636	***PA4923***	195	1_Pseudomonas_aureginosa
G0EZM0	***CNE_1c09680***	194	1_Ralstonia_eutropha
P47044	***YJL055W***	245	1_Saccharomyces_cerevisiae
Q84MC2	***Log8/At5g11950***	216	1_Arabidopsis_thaliana_AtLOG8_1YDH
M1VUY5	***CPUR_02269***	240	1_Claviceps_purpurea_CpLOG_5AJT
Q5ZC82	***log/Os01g0588900***	242	1_Oryza_sativa_OsLOG
O05306	***Rv1205***	187	1_Mycobacterium_tuberculosis_MtLOG
B2HS63	***MMAR_4233***	187	1_Mycobacterium_marinum_MmLOG_3SBX
I7ICL1	***log1***	226	1_Eleusine_coracana_(finger_millet)
O06986	***yvdD/BSU34640***	191	1_Bacillus_subtilis_168_1T35_YvdD
F1TJI9	***HMPREF0724_13892***	186	1_Rhodococcus_equi
U5ECZ6	***NCAST_23_00810***	186	1_Nocardia_asteroides
V8CS24	***W823_26605***	179	1_Williamsia_sp.D3
R7YE42	***GTC6_04665***	178	1_Gordonia_terrae_C-6
D5UVY6	***Tpau_1162***	182	1_Tsukamurella_paurometabola_DSM20162
E6JCG2	***ES5_14648***	184	1_Dietzia_cinnamea_P4
A0A0M4CHB3	***CDES_10825***	198	1_Corynebacterium_deserti
A4F8H8	***SACE_1021***	183	1_Saccharopolyspora_erythraea_NRRL2338
F4CMC7	***Psed_1016***	195	1_Pseudonocardia_dioxanivorans_CB1190
C6WKY2	***Amir_0775***	180	1_Actinosynnema_mirum_DSM43827
A0A099D0Q3	***IL38_22405***	175	1_Actinopolyspora_erythraea
A0A109IIT8	***AWV63_18275***	181	1_Micromonospora_rifamycinica
D2B0Q9	***Sros_8427***	195	1_Streptosporangium_roseum
A0A132N4S9	***TH66_04050***	197	1_Streptomyces_thermoautotrophicus
Q0RCV1	***FRAAL6099***	211	1_Frankia_alni_ACN14a

Supplementary Figure S3. List of LOG proteins used for phylogenetic tree analysis.

Uniprot (Ncbi) Accession Code	Gene name or Locus name	Amino acids	Assignment
D6Y7L0	***Tbis_3024***	191	1_Thermobispora_bispora_DSM43833
A0A0N9JLQ5	***AOZ07_01285***	199	1_Arthrobacter_arilaitensis
A0A0W7W772	***AUL38_13360***	206	1_Leucobacter_sp.G161
P46378	***pFiD188 fas6***	198	1_Rhodococcus_fascians_D188_plasmid
Q5IK40	***fas6/stPAI019***	191	1_streptomyces_graminilatus
A0A0C1WGC2	***QH73_40975***	194	1_Scytonema_millei_VB511283
A0A0C2Q1Q0	***SD81_13890***	194	1_Tolypothrix_campylonemoides_VB511288
B8HZF0	***Cyan7425_0098***	193	1_Cyanothece_sp.PCC
D7BGC7	***Mesil_1866***	193	1_Meiothermus_silvanus_DSM9946
B9L3L3	***trd_A0376***	201	1_Thermomicrobium_roseum_DSM5159_plasmid
D6TLP5	***Krac_8006***	197	1_Ktedonobacter_racemifer_DSM44963
A0A063XZJ7	***ADINL_1957***	186	1_Nitrincola_lacisaponensis
F6CT79	***Mar181_3222***	186	1_Marinomonas_posidonia
S7Y353	***L292_0777***	194	1_Acinetobacter_junii_CIP107470
N9DLT9	***F941_02996***	198	1_Acinetobacter_bouvetii
A6EYE2	***MDG893_18447***	186	1_Marinobacter_algicola
S0AI08	***SOD_c25560***	190	1_Serratia_plymuthica
A0A0H3FS17	***EAE_17755***	189	1_Enterobacter_aerogenes_KCTC2190
A0A085GT43	***GHAL_2696***	189	1_Hafnia_alvei_ATCC13337
A0A087L459	***IV04_06625***	190	1_Serratia_sp.Ag1
A0A168M9A7	***SAMEA2273715_01144***	192	1_Klebsiella_pneumoniae_UHKPC81
A0A085HL15	***GLGR_1022***	191	1_Leminorella grimontii
I3CKR8	***BegalDRAFT_3393***	194	1_Beggiatoa_alba
WP_020560233 .1 (Ncbi refseq)	-	193	1_Thiothrix_flexilis
A0A0U3AMY4	***AXO1947_07520***	197	1_Xanthomonas_oryzae
WP_051298963 .1 (Ncbi refseq)	-	193	1_Marinobacterium_litorale
U2EQZ1	***SSPSH_000572***	193	1_Salinisphaera_shabanensis_E1L3A

Supplementary Figure S3. List of LOG proteins used for phylogenetic tree analysis. (continued)

Uniprot (Ncbi) Accession Code	Gene name or Locus name	Amino acids	Assignment
Q7NVI6	***CV_2356***	212	1_Chromobacterium_violaceum_ATCC12472
U1AWK6	***O166_06975***	196	1_Pseudogulbenkiania_ferrooxidans
A0A070A2X1	***GLUCORHAEAF1_11940***	191	1_Komagataeibacter_rhaeticus
E3I6I0	***Rvan_0251***	193	1_Rhodomicrobium_vannielii
WP_020590295 .1 (Ncbi refseq)	*-*	179	1_Desulfobacter curvatus
A0A084T1P4	***Q664_01610***	198	1_Cystobacter_violaceus
Q1D3H6	***MXAN_4631***	197	1_Myxococcus_xanthus
A0A0D5N4I2	***RW64_05155***	196	1_Geobacter sulfurreducens
WP_044415183 .1 (Ncbi refseq)	*-*	186	1_Arcobacter_anaerophilus
WP_024955895 .1 (Ncbi refseq)	*-*	185	1_Sulfurospirillum_arcachonense
WP_044934207 .1 (Ncbi refseq)	*-*	205	1_Acidobacterium_sp.PMMR2
A0A0P0CS10	***DC20_10035***	193	1_Rufibacter_tibetensis
WP_050060133 .1 (Ncbi refseq)	*-*	202	1_Silvibacterium_bohemicum
A6CFE1	***PM8797T_14609***	196	1_Gimesia_maris
D2R366	***Psta_0407***	202	1_Pirellula_staleyi
Q0W2V0	***RCIX2172***	200	1_Methanocella_arvoryzae
Q8NRE2	***Cg1261/Cgl1110***	256	2_Corynebacterium_glutamicum_CgLOGII
Q6NHZ7	***DIP0983***	254	2_Corynebacterium_diphtheriae_ATCC700971
C1A2P3	***RER_41700***	267	2_Rhodococcus_erythropolis_PR4
A0A165M823	***A3L23_03098***	256	2_Rhodococcus_fascians_D188_typeII
A0A0B8N681	***NS07_v2contig00049-0007***	258	2_Norcadia_seriolae
A0A0E3XNJ5	***GR01_06160***	251	2_Mycobacterium_chelonae
A0A024M085	***BN975_02456***	245	2_Mycobacterium_farcinogenes
E6JCG1	***ES5_14643***	266	2_Dietzia_cinnamea_P4
E8WES6	***Sfla_2156***	261	2_Streptomyces_pratensis_ATCC33331
A0A093AR08	***BB31_40960***	261	2_Amycolatopsis_lurida_NRRL2430
C7MVI2	***Svir_06300***	260	2_Saccharomonospora_viridis_DSM43017

Supplementary Figure S3. List of LOG proteins used for phylogenetic tree analysis. (continued)

Uniprot (Ncbi) Accession Code	Gene name or Locus name	Amino acids	Assignment
A0A0F0H3D1	***UK23_17865***	261	2_Lechevalieria_aerocolonigenes
Q2J6A1	***Francci3_3841***	258	2_Frankia_sp_CcI3
D6Y7K9	***Tbis_3023***	248	2_Thermobispora_bispora_DSM43833
Q5SHT6	***TTHA1644/Tt1465***	217	2_Thermus_thermophilus_TtLOGII_1WEK
E4U4W4	***Ocepr_1862***	222	2_Oceanithermus_profundus_DSM14977
D9XE68	***SSQG_05174***	252	2_Streptomyces_viridochromogenes_DSM40736
A0A0Q8P137	***ASE03_03545***	268	2_Kitasatospora_sp_Root187
A0A0N7FAX8	***AOZ07_11110***	257	2_Glutamicibacter_arilaitensis
W2EHT4	***MPTA5024_36350***	255	2_Microbispora_sp_ATCC_PTA-5024
H5U3J5	***GOSPT_098_00080***	287	2_Gordonia_sputi_NBRC100414
H6RUD6	***BLASA_0951***	275	2_Blastococcus_saxobsidens_DD2
Q2LWS8	***SYN_01766***	219	2_Syntrophus_aciditrophicus_SB
E8RAS4	***Despr_0390***	223	2_Desulfobulbus_propionicus_DSM2032
F9ZXY6	***Metme_0115***	238	2_Methylomonas_methanica_MC09
D3SBR4	***TK90_0045***	241	2_Thioalkalivibrio_sp_K90mix
A4BNY3	***NB231_10778***	244	2_Nitrococcus_mobilis_Nb-231
M7NXX7	***MPL1_11950***	234	2_Methylophaga_lonarensis_MPL
A0A0S7YKB6	***AMS14_04130***	233	2_Planctomycetes_bacterium_DG_20
C4FJX0	***SULYE_0870***	225	2_Sulfurihydrogenibium_yellowstonense_SS-5
Q0YUG1	***CferDRAFT_2077***	245	2_Chlorobium_ferrooxidans_DSM13031
Q9FBL8	***SCO5140***	252	2_Streptomyces_coelicolor_A3
D2PN34	***Kfla_5508***	288	2_Kribbella_flavida_DSM17836
E4NGJ5	***KSE_48470***	260	2_Kitasatospora_setae_ATCC33774
Q82IK9	***SAVERM_3124***	252	2_Streptomyces_avermitilis_ATCC31267
A0A076MJU3	***AMETH_0942***	256	2_Amycolatopsis_methanolica_239
Q93XW9	***At1g50575***	306	2_Arabidopsis_thaliana_typeII
B7FM71	***MTR_3g055920***	312	2_Medicago_truncatula(Barrel_medic)

Supplementary Figure S3. List of LOG proteins used for phylogenetic tree analysis. (continued)

Uniprot (Ncbi) Accession Code	Gene name or Locus name	Amino acids	Assignment
M5WGN8	***PRUPE_ppa009177mg***	303	2_Prunus_persica(Peach)
Q6F2U6	***LOC_Os03g39010***	335	2_Oryza_sativa(rice)_typeII
B2GKP8	***KRH_08300***	292	2_Kocuria_rhizophila_ATCC9341
D7CTR8	***Trad_2507***	234	2_Truepera_radiovictrix_DSM17093
A0A106BQK1	***ABW22_07560***	242	2_Thiobacillus_denitrificans
A0A177MB76	***A1332_03115***	234	2_Methylomonas_methanica
G4SZU5	***MEALZ_3889***	242	2_Methylomicrobium_alcaliphilum
E1JRJ3	***DesfrDRAFT_0242***	218	2_Desulfovibrio_fructosivorans_JJ
A0A0P0U5S9	***AMR72_00800***	240	2_Flavobacterium_psychrophilum
A5FBB5	***Fjoh_4510***	242	2_Flavobacterium_johnsoniae_ATCC17061
A0A0C1FQT0	***OA86_05045***	239	2_Chryseobacterium_jeonii
R9CQU8	***L100_00200***	243	2_Elizabethkingia_meningoseptica_ATCC13253
L7WI78	***DDD_3581***	229	2_Nonlabens_dokdonensis_DSM17205
K1LAV5	***B879_02032***	244	2_Cecembia_lonarensis_LW9
L8JKS3	***C900_04919***	262	2_Fulvivirga_imtechensis_AK7

Supplementary Figure S3. List of LOG proteins used for phylogenetic tree analysis. (continued)
